# Skin Barrier-Enhancing Effects of Dermabiotics HDB with Regulation of Skin Microbiota

**DOI:** 10.4014/jmb.2306.06042

**Published:** 2023-10-19

**Authors:** Kyung Min Kim, Ji-Won Song, Chang-Wan Lee, Du-Seong Kim, Johann Sohn, Seunghun Lee

**Affiliations:** Biohealthcare R&D Center, HYUNDAI BIOLAND Co., Ltd., Ansan 15407, Republic of Korea

**Keywords:** Probiotics, cell lysate, skin moisturizing, skin microbiota

## Abstract

In the regulation of inflammatory responses and skin homeostasis, the skin and its microbiota are closely related. Studies have reported that lactic acid bacteria extracts can improve the skin condition and microbiota. In our previous study, we developed probiotic lysates, which are efficacious in improvement of human skin cells and the skin barrier. The skin-moisturizing effect of Dermabiotics HDB (HDB) prepared with *Lactiplantibacillus plantarum*, and the correlation between changes in the skin microbiota and moisture contents, were evaluated and analyzed in clinical trials. The clinical parameters on the cheeks of 21 female participants were measured using biophysical tools before and after (2 weeks) using HDB or control. The skin microbes were collected and identified using 16s rRNA gene sequencing. HDB significantly improved moisture intensity, transepidermal water loss (TEWL), and hot flush level on the cheek. The beta-diversity of the skin microbiota was different from that of the control in the unweighted UniFrac principal coordinate analysis after using HDB. The genus *Lawsonella* demonstrated a positive correlation with TEWL and a negative correlation with the moisture contents of the keratin layer, regardless of the use of HDB and control. Conversely, after HDB use, the genus *Staphylococcus* was increased and associated with a lower hot flush level, while the genera of the phylum Proteobacteria tended to decrease, which is associated with an improved skin condition. Overall, HDB showed clinically proven effects, including skin moisturization with regulation of the skin microbiota.

## Introduction

As the largest organ of the human body, the skin is a defensive barrier that protects the internal matrix from harmful elements of the external environment, such as ultraviolet radiation, infectious pathogens, or chemical pollutants [1]. The skin is home to a complex ecosystem inhabited by various microorganisms, including bacteria, fungi, and viruses. The skin and its microbiota are closely interlinked in the regulation of inflammatory responses and homeostasis, as well as skin acidity, the permeation of substances, and defense against the invasion of pathogens [2, 3]. Many reports suggest that a dysbiosis between skin commensal bacteria and invasive pathogens can lead to various skin disorders [3, 4]. *Staphylococcus epidermidis* usually plays a role in inhibiting skin infections secondary to *Staphylococcus aureus*; however, when the composition of *S. aureus* increases under certain conditions, it may opportunistically act as a pathogen and cause atopic symptoms [5]. In addition, *Cutibacterium acne* is predominantly localized to the sebaceous glands, and in acne-induced skin, the ratio is particularly high [6, 7]. Meanwhile, the mechanism of interaction between the clinical symptoms of the skin and the microflora remains unclear.

Recently, lactic acid bacteria extracts have been used to improve skin conditions. Heat-killed *Lactobacillus acidophilus* KCCM12625P possesses anti-wrinkle and whitening effects in UVB-irradiated cells [8]. *Lacticaseibacillus rhamnosus* and *Limosilactobacillus reuteri* DSM 17938 lysates improved the skin barrier function and hydration effects in reconstructed human epidermis [9, 10]. Particularly, *L. reuteri* DSM 17938 inhibited the growth of pathogenic skin bacteria *Pseudomonas aeruginosa* and *Streptococcus pyogenes*. Furthermore, *Lactiplantibacillus plantarum* APsulloc 331261 fermented lysate has regulatory activity on the microorganisms known as commensal skin microflora, *S. epidermidis* and *S. aureus* [11]. Although probiotic lysates can control the microflora, further research is still warranted to identify the active components.

In our previous study, probiotic lysates with skin-moisturizing efficacy on the human skin cells were developed and reported [12]. This clinical trial was conducted to evaluate the effects of previously identified *L. plantarum* HDB on skin barrier enhancement and skin microbiota regulation. Its potential as a skin microbiome-based cosmetic ingredient was confirmed through a correlation analysis between changes in skin microbiota and clinical data.

## Materials and Methods

### Probiotic Lysate Preparation

*L. plantarum* HDB was provided by the HDB Cell Bank (Hyundai Bioland Co., Ltd., Korea) and grown in de Man, Rogosa, and Sharpe (MRS; BD BBL, Franklin Lakes, NJ, USA) broth at 37°C for 24 h. Overnight cultures were centrifuged at 20,000 ×*g* for 10 min and washed twice with distilled water. The harvested cells were inoculated into distilled water and responding cells were disrupted by heating at 121°C and 0.12 MPa for 2 h. After purification with a 0.45-μm filter to remove the cell wall, the prepared lysate was named HDB and used in subsequent experiments. For the clinical study, a general essence cosmetic sample without HDB and consisting of glycerin, butylene glycol, polyethylene glycol, oils, and preservative, was used as a control. Also, by adding 3% (v/v) of HDB to the same composition, a test sample was prepared.

### Clinical Study Design

This study was approved by the Institutional Review Board (Approval No. 2020021201-202203-HR-001-01) and all volunteers participated in the test after signing the test consent form. The study was conducted in compliance with ethical regulations and bioethics according to the Declaration of Helsinki. A total of 21 healthy Korean female volunteers (20–50 years in age) participated in this study. The exclusion criteria are as follows: 1) pregnant or lactating; 2) with infectious or atopic skin disorder; 3) has sensitive and hypersensitive skin; 4) had intake of steroids to treat skin disorder; 5) and participated in a similar study within 3 months. Both sides of the cheek were the test site, and the participants were provided with cosmetics randomly assigned to the corresponding site. The participants applied the test (Essence with 3% HDB) and the control sample (Essence without Dermabiotics HDB, Control) to the corresponding area twice a day for 2 weeks. Before use (0 week) and 2 weeks after use, clinical evaluation was conducted and skin samples were taken after the participant rested for at least 15 min under constant temperature and humidity conditions (22°C ± 2°C and 50% ± 10% relative humidity), respectively.

### Measurement of Skin Parameters

By obtaining a moisture map image of the test site, moisture intensity was evaluated using an Epsilon E100 (Biox Systems Ltd., UK), and a three-dimensional image was rendered. Tape stripping was performed using D-Squame sampling disks (D100, Clinical and derm) at each evaluation time point before and after use of the cosmetic sample (2nd week of use), and the disks were used for skin microbiota analysis. Transepidermal water loss (TEWL) was measured using Vapometer (Delfin, Finland), and skin redness was measured using Image J software (NIH, USA) after taking pictures of the face using VISIA-CR (Canfield Scientific, USA).

### Skin Microbiota Analysis

**Library Preparation and Sequencing**

Using the AccuStool DNA Preparation Kit (AccuGene, Korea), DNA extraction from skin microbiota samples was performed in accordance with the manufacturer’s instructions. The hypervariable V4 region of the 16S rRNA gene was amplified from the DNA extracts through 25 PCR cycles using KAPA HiFi HotStart ReadyMix (Roche, Swiss) and barcoded fusion primers 515fb/806rb containing Nextera adaptors. PCR products (~250 bp) were purified with HiAccuBeads (AccuGene) [13]. The amplicon libraries were pooled at an equimolar ratio and the pooled libraries were sequenced on an Illumina MiSeq system using MiSeq Reagent Kit v2 for 500 cycles (Illumina, USA). The sequencing data of 16S rRNA genes are publicly available in the NCBI Short Read Archive under accession number SRP428383 (NCBI BioProject PRJNA946350).

### Data Analysis

For all raw data sets, VSEARCH v2.10.3 was used to remove chimeric 16S rRNA gene sequences from filtered reads [14]. Using the QIIME 1.9.1 software package (http://qiime.org), downstream analyses of quality and chimera filtered reads were performed [15]. Each of the quality-filtered sequencing read datasets was assigned to operational taxonomic units with a threshold of 97% pairwise identity using QIIME’s reference-based workflow scripts and the SILVA release 132 rRNA reference database. Principal coordinate analysis (PCoA) plots based on the unweighted UniFrac were generated using the QIIME package (v1.9.1) and were used to determine the similarity between different skin microbiota samples based on microbial compositions in the dataset. Canonical correspondence analysis (CCA) plots were constructed using SciKit-Learn version 0.19.1 and were used to visualize the relationship of the skin microbiota with specific parameters related to skin conditions.

### Statistical Analysis

All results are presented as mean ± SD of at least three experiments. The Wilcoxon signed-rank test or paired *t*-test was used to compare the before-and-after samples. In PCoA and microbiota analysis, the control and HDB groups were compared using an independent *t*-test or Mann–Whitney U test of SPSS Static 19.0 (SPSS, Inc., USA). Correlation analysis between changes in microbiota and changes in clinical parameters were performed using Pearson correlation analysis. Statistical significance was set at *p* < 0.05.

## Results

### Clinical Effects of HDB

**Fig. 1:**
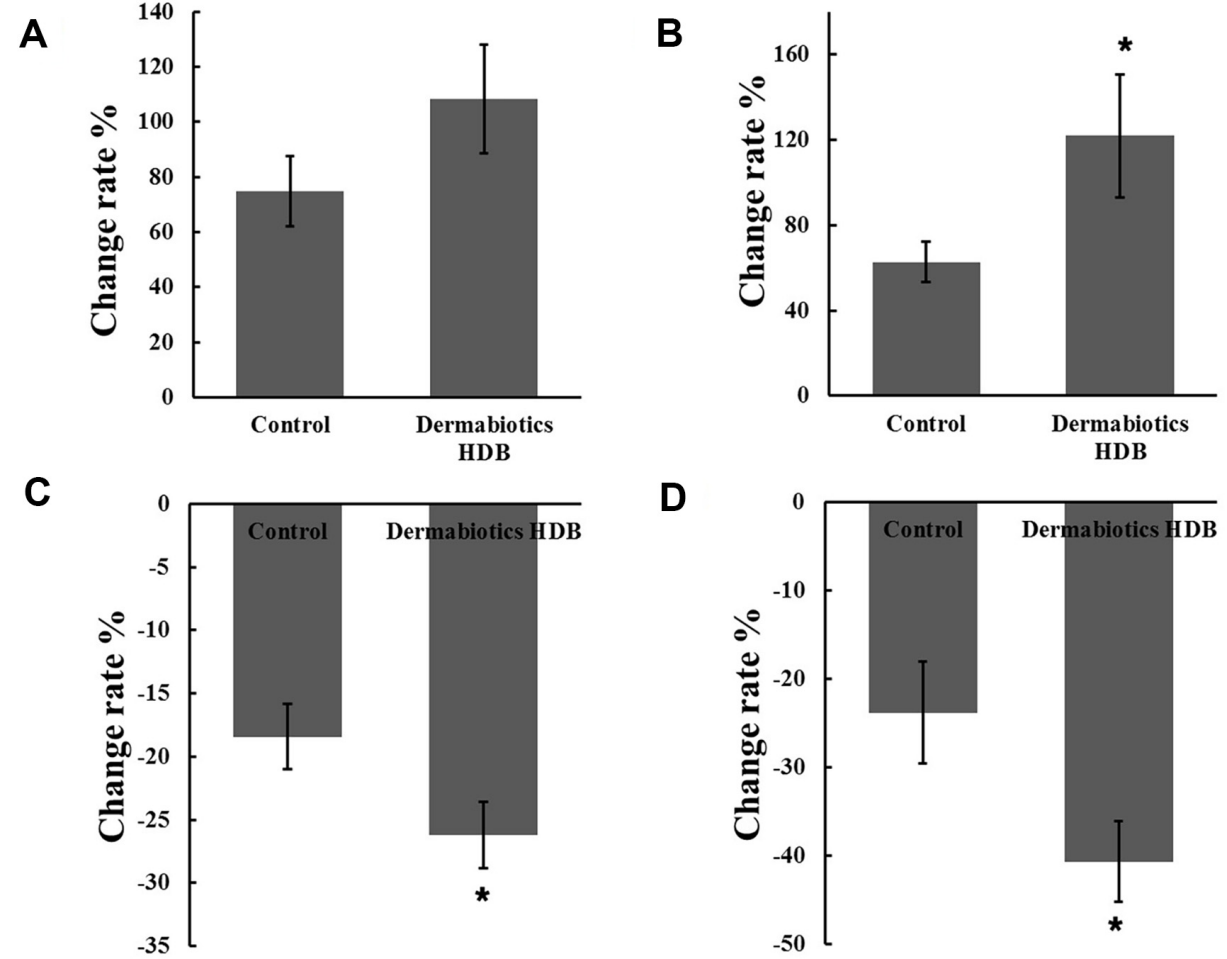
Comparison of clinical parameter changes after using Dermabiotics HDB (HDB). Moisture intensity of the keratin layer surface (**A**) and keratin layer (**B**). (**C**) Transepidermal water loss. (**D**) Hot flush level. The data are shown as mean ± SD of three independent experiments. *p < 0.05, vs. control.

Improvement in all clinical parameters (Table 1) was observed after a 2-week use in both groups (control and HDB), and particularly, an increase in moisture intensity of the keratin layer was noted after using HDB. The HDB group was noted to have increased water intensity of the keratin layer surface and inside by 108.29% and 121.83%, respectively, while the control increased by 71.40% and 49.45%, respectively (Fig. 1A and 1B). Conversely, TEWL and flushing levels were significantly reduced in both groups; however, when HDB was used, the improvement increased by 7.82% and 16.81% on average, respectively (Fig. 1C and 1D).

### Effects on the Skin Microbiota

**Fig. 2:**
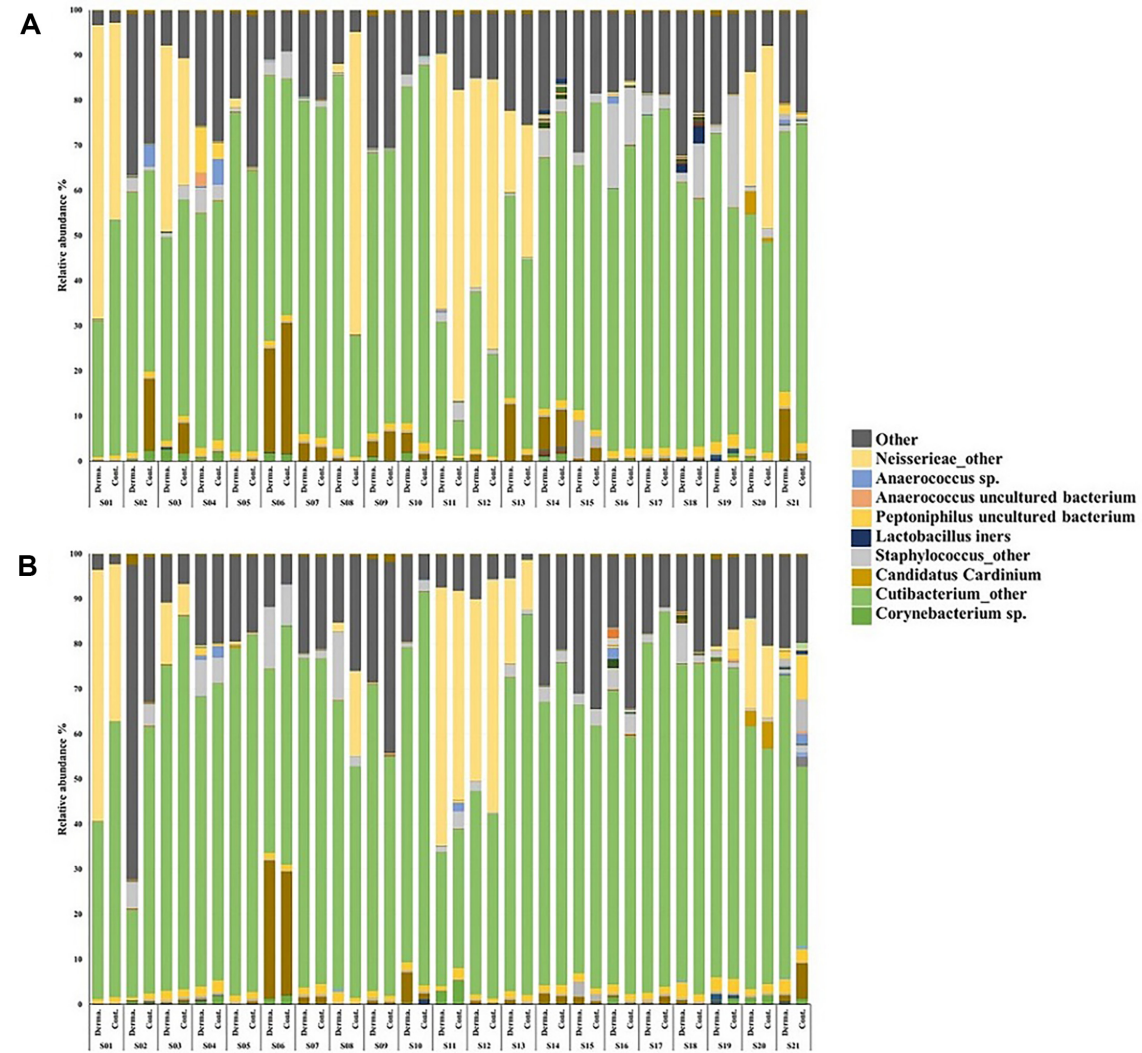
Comparison of abundance of skin microbiota in each participant before (A) and after (B) using cosmetics.

**Fig. 3:**
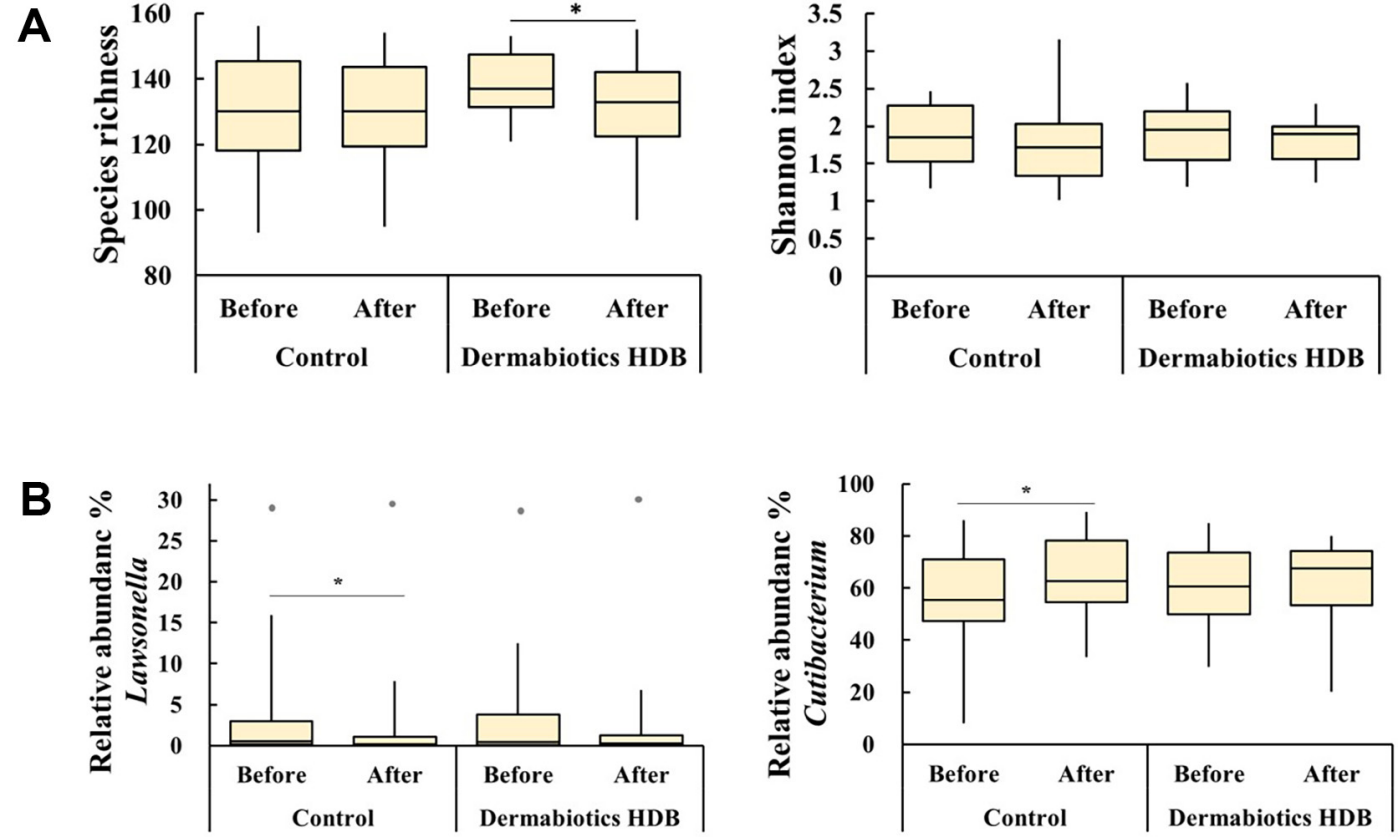
Comparison of the diversity of the skin microbiota of HDB and the control group. Alpha-diversityspecies richness (**A**) and Shannon index (**B**) Comparison of abundance of major microbes *Lawsonella* (**C**) and *Cutibacterium* (**D**). The data are shown as mean ± SD of three independent experiments. Wilcoxon signed-rank test was used for statistical analysis. *p < 0.05, vs. control.

Before sample use (week 0), the composition of the skin microbiota of each participant was very diverse. Moreover, the composition of the analyzed microbiota from both cheeks of each participant was different from each other (Fig. 2A). At 2 weeks of sample use (Fig. 2B), the composition of the skin microbiota was not similar. As a result of α-diversity analysis, species richness was significantly decreased after HDB treatment; however, no significant difference in the Shannon index was noted (Fig. 3A). Among the skin commensal bacteria, the genus *Lawsonella*, suborder of *Corynebactericeae*, tended to decrease after use of both samples; however, the genus *Cutibacterium* increased after use of control (Fig. 3B).

### Correlation Between Skin parameters and Microbiota Shift

**Fig. 4:**
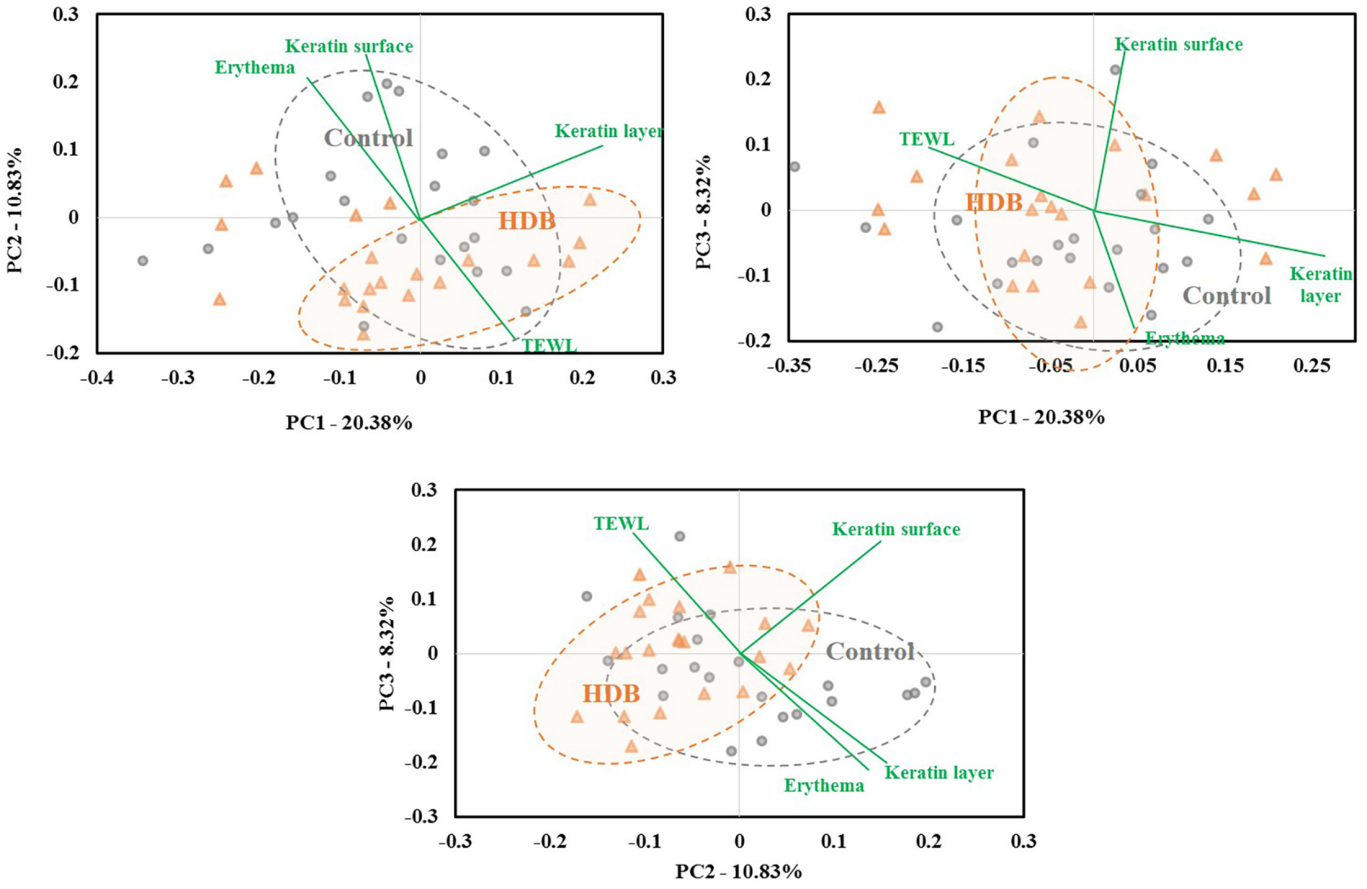
β-diversity with principal coordinate analysis (PCoA) plot of unweighted UniFrac distances and canonical correspondence analysis. Gray circles and orange triangles represent control and HDB after use each.

**Fig. 5:**
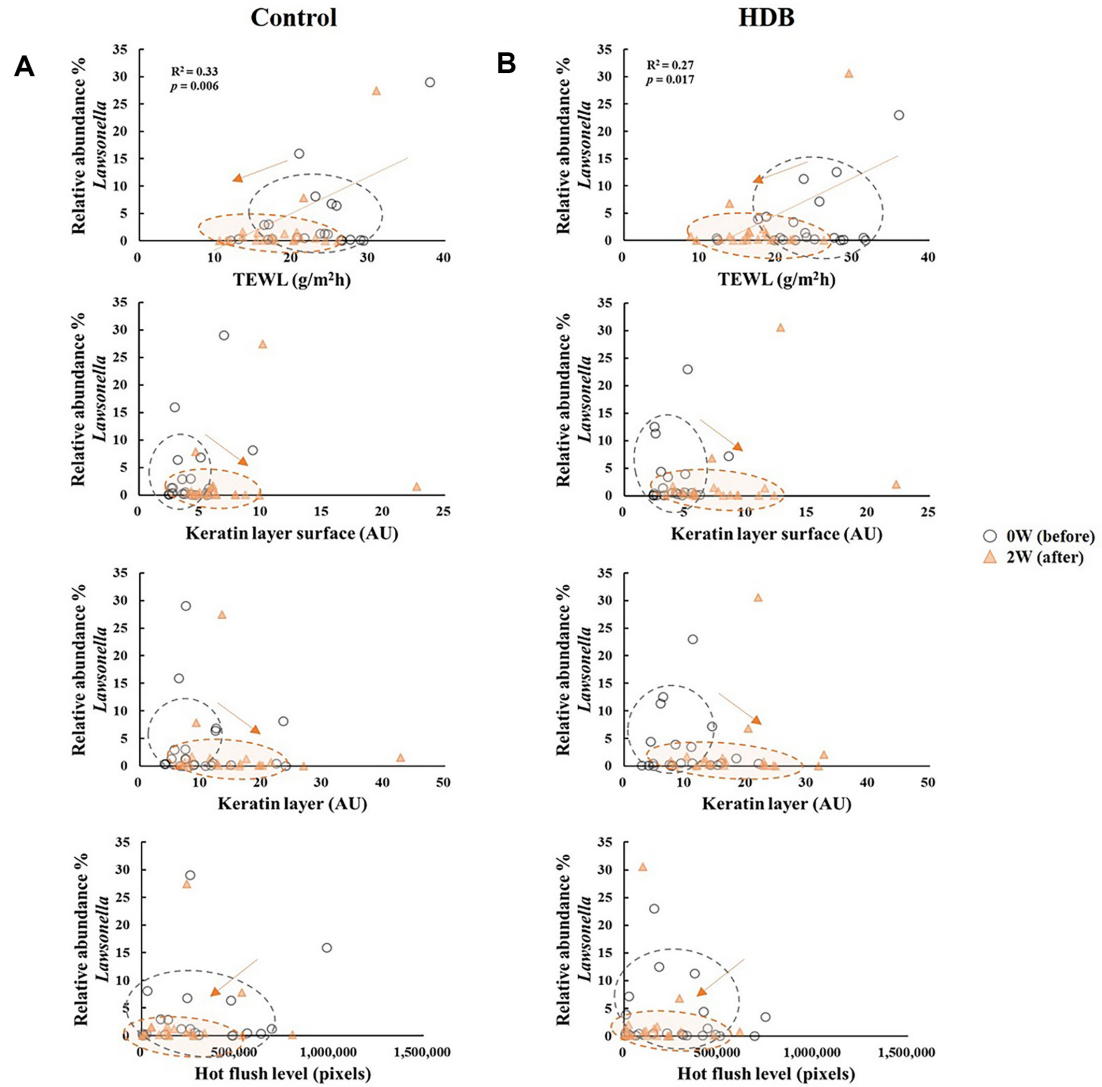
Correlation analysis between the relative abundance (%) of the genus *Lawsonella* and clinical skin parameters of control (A) and HDB (B).

**Fig. 6:**
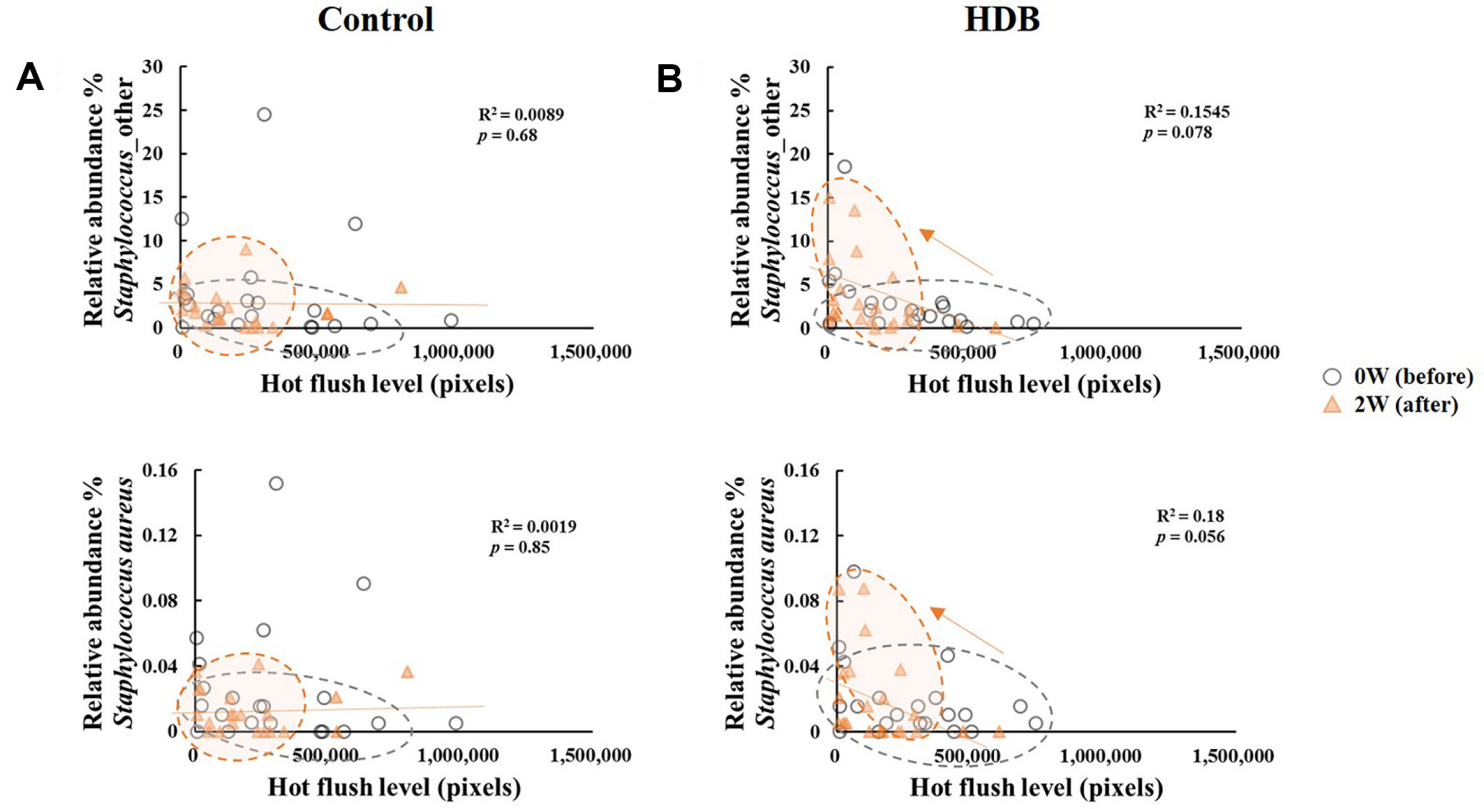
Correlation analysis between the relative abundance (%) of the genus *Staphylococcus* sp. and *S. aureus* clinical skin parameters of control (A) and HDB (B).

**Fig. 7:**
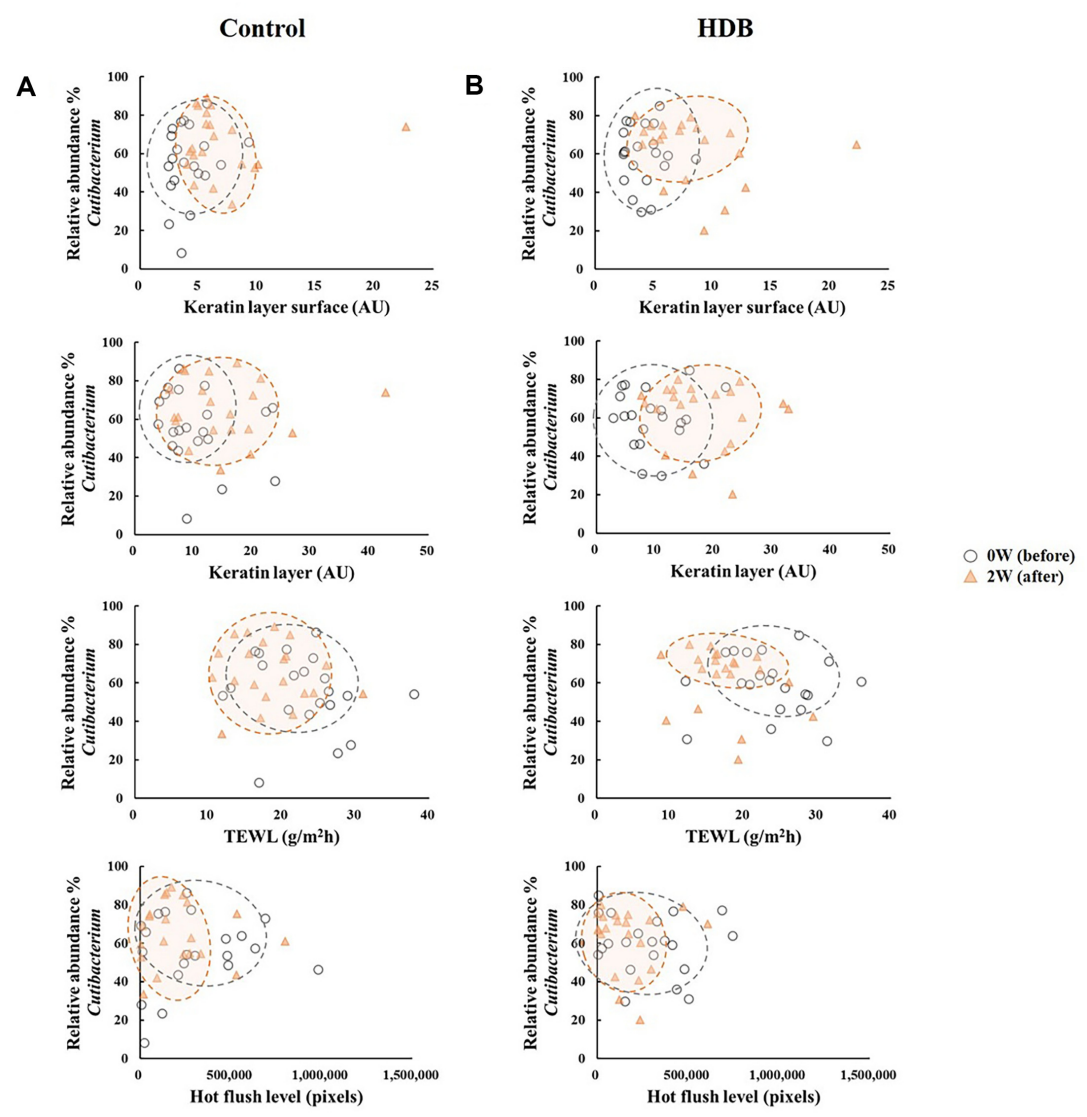
Correlation analysis between the relative abundance (%) of the genus *Cutibacterium* clinical skin parameters of control (A) and HDB (B).

As a result of the β-diversity with unweighted UniFrac PCoA, the shift in skin microbiota after using HDB showed a tendency to be grouped separately from those of the control (*p* < 0.05 for PC2, Fig. 4). In the CCA, TEWL vector tended to be drawn along the PC2 axis, and the HDB group plots were concentrated in the TEWL direction. Therefore, it is predicted that a correlation between skin microbiota of the HDB group and TEWL exists. In the correlation analysis, *Lawsosnella*, which has a decreasing trend in both groups, showed a proportional relationship to TEWL (*p* < 0.05, Fig. 5). Additionally, an inverse relationship with moisture content in the keratin layer was observed regardless of whether both groups were used before or after, which showed no correlation with the flushing levels. *Staphylococcus* including *S. aureus* was inversely correlated with the hot flushing level of the cheeks in HDB only (Fig. 6). In contrast, *Cutibacterium* showed no correlation with any of the clinical parameters (Fig. 7).

**Fig. 8:**
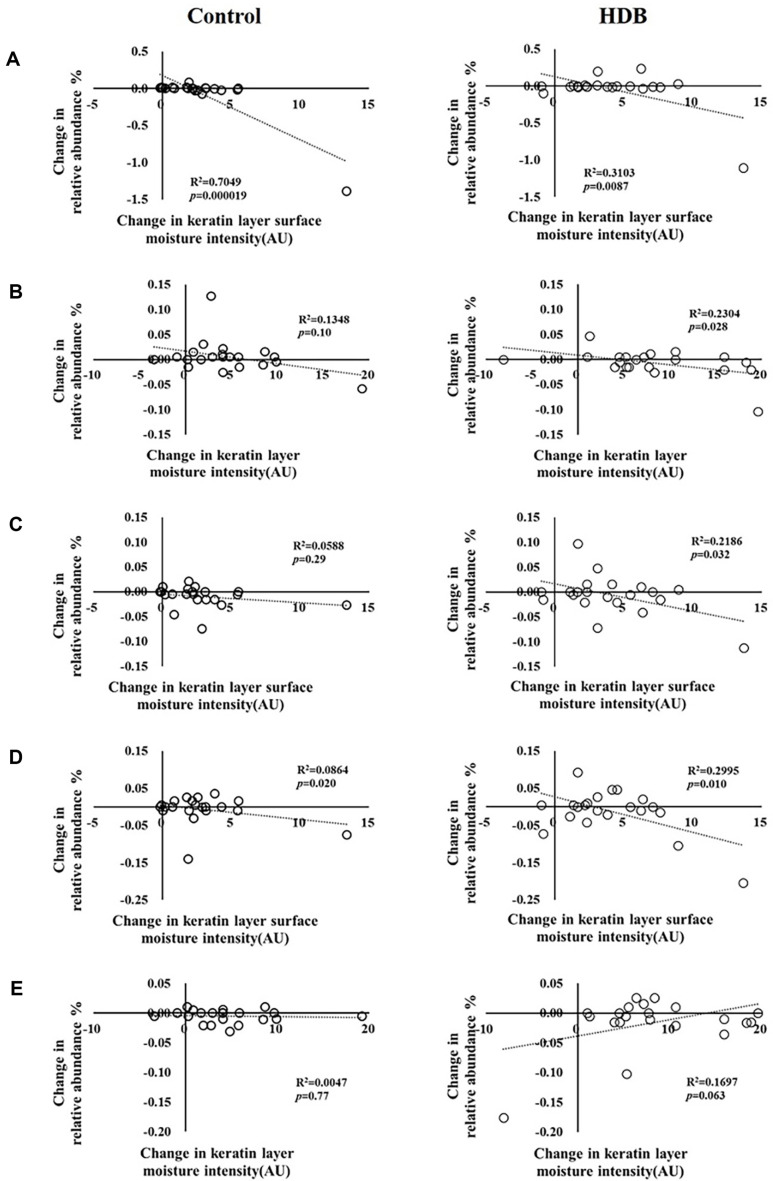
Correlation between changes in microflora at the genus level and changes in clinical parameters after using control (left) and HDB (right). (**A**) *Corynebacterium*, (**B**) *Halomonas*, (**C**) *Marinobacter*, (**D**) *Enhydrobacter*, and (**E**) *Cloacibacterium*.

An analysis was conducted on the correlation between the amount of change in clinical indicators and in the skin microbiota before and after use of cosmetics. The genus *Corynebacterium* decreased inversely in proportion to the increase in water density of the keratin layer surface of the cheeks, regardless of HDB and control (Fig. 8A). The genera *Halomonas*, *Marinobacter*, and *Enhydrobacter* belonging to the gamma-Proteobacteria class were significantly decreased after using HDB with an inverse correlation with the increased amount of moisture intensity (Fig. 8B-8D). Conversely, the genus *Cloacibacterium* revealed a correlation that increased in proportion to the increase in moisture intensity according to the use of HDB (Fig. 8E).

## Discussion

The skin microbiota are very different for each individual, and depending on the topographical location, the composition of the microbes for each body part is variable even within the same individual [2]. According to a study, even if the same moisturizer is used on skin representing different microbiota, it is very difficult for the final microbiota composition to be similar among individuals [16]. In this study, a clinical trial was conducted using HDB, a lactic acid bacteria lysate as a cosmetic ingredient. Owing to the differences in the composition at the starting point for each participant, a bias may occur in the data for skin microbiota changes. The cheek, which takes up the largest area of the human face and can be accurately divided into two sites, was selected as the cosmetic sample application area, and both cheeks of each participant were randomly divided into a test and control group. Before the sample was used, there was a difference in the microbiota of both cheeks of each participant; however, the diversity was remarkably lesser compared to the composition of the microbiota between individuals, which would be more advantageous for a comparison between the test and control group (Fig. 2). Conversely, it has been reported that differences exist in the skin microbiota analysis results depending on the sampling method [17]. A tool that can accurately collect skin microbiota to conduct a full analysis has yet to be developed.

In the clinical results, when HDB was used, a significant improvement in the clinical indicators was observed compared to control. Both TEWL and moisture intensity of the keratin layer surface, which are the most basic indicators of skin health, were improved (Fig. 1). Particularly, in the CCA analysis, when the relationship between each variable and the clinical parameters was estimated, clustering of only the HDB plot, which was distinct from the plot of the control sample, was confirmed. TEWL revealed a correlation after using HDB in the beta-diversity analysis of the microbiota data (Fig. 4). These results indicate that HDB can consistently induce changes in the skin microbiota. At the genus level, *Lawsonella* has been known as a species of skin microbiota that correlates with skin moisture content [6, 18]. *Lawsonella clevelandensis* was reported to show an inverse relationship to cheek moisture density, and *Corynebacterium*, a member of the same order, was proportional to TEWL. Regardless of whether or not HDB was used, this experiment also showed a proportional correlation with TEWL and an inversely proportional correlation with the keratin layer surface water intensity (Fig. 5). In the future, the relationship between these microbes and skin moisture content should be explored in detail through more research and big data construction.

The genus *Staphylococcus*, one of the dominant skin genera, was more dominant in sebaceous glands or areas with moisture than in dry areas [2]. It showed an inversely proportional tendency in which hot flush symptoms decreased after using HDB and the composition increased (Fig. 6). However, after using the control sample, this correlation was not demonstrated and only the clinical symptoms were alleviated. *Staphylococcus* includes *S. epidermidis* as a skin dominant bacterium, and this microbe is known to play a role in controlling the growth of *S. aureus* and maintaining skin homeostasis [5]. *S. aureus* is a species of pathogenic bacteria that opportunistically increases, penetrates into the sebaceous glands and stratum corneum, and induces an inflammatory response and psoriasis [6]. In patients with atopic dermatitis, *S. aureus* colonizes the skin with a frequency of more than 80%. In this study, the ratio of *S. aureus* was detected as a non-dominant microbiota at 1% in the total ratio of the genus *Staphylococcus*, both before and after using the cosmetic. A hot flush in the skin, also known as rosacea, is a phenomenon that appears prominently due to inflammation or blood vessel expansion as the skin barrier collapses and sensitivity to external stimuli increases [19]. Interest in the role of the microbiome in the onset and exacerbation of rosacea continues to grow. HDB probably increases the composition of dominant species, which likely modulates the microbiome composition as a skin barrier by controlling skin pathogens.

It has been reported that the phylum Proteobacteria dominates the dry areas of the skin [1, 20, 21]. In particular, class beta and gamma-Proteobacteria, especially genus *Enhydrobacter*, tend to dominate in dry areas [1, 21]. Conversely, the family *Flavonobacterials* dominates in both high moisture and dry areas, but is very rare in sebaceous glands [21]. After the use of HDB, the reduction of genera *Halomonas*, *Marinobacter*, and *Enhydrobacter*, belonging to gamma-Proteobacteria, and the increase in the genus *Cloacibacterium*, belonging to *Flavonobacteriales*, were correlated with an increase in water intensity of the keratin layer surface (Fig. 8). HDB did not render the same composition of the final skin microbiota, and this may be attributed to the fact that each participant had varying compositions in their skin microbiota prior to the cosmetic use. However, changes in the increase or decrease of bacteria correlated with skin moisture content were confirmed. These results indicate that HDB can be a candidate material for enhancing the protective mechanism of the complex skin barrier through regulation of not only skin moisture but also the abundance of microbiota. However, the mechanism of the interaction between skin microbes and clinical biomarkers remains poorly understood. In the future, securing a microbiome database through more clinical trials to identify the correlation between the skin microbiome and clinical indicators, including mechanism of action, will be very useful.

## Supplemental Materials

Supplementary data for this paper are available on-line only at http://jmb.or.kr.



## Figures and Tables

**Table 1:** The results of the clinical test of Dermabiotics HDB.

Sample		Moisture intensity (AU)				TEWL (g/m^2^h)		Hot flush level (pixels)	
		Keratin layer surface		Keratin layer					
		Average	*p*-value	Average	*p*-value	Average	*p*-value	Average	*p*-value
Control	0 week	4.16 ± 0.38	-	10.66 ± 1.31	-	22.82 ± 1.33	-	300793.81 ± 57907.06	-
	2 weeks	6.93 ± 0.88	<0.001	15.19 ± 1.85	<0.001	18.44 ± 1.12	<0.001	212504.67 ± 44451.02	<0.001
Dermabiotics HDB	0 week	4.12 ± 0.35	-	9.89 ± 1.13	-	23.78 ± 1.31	-	282460.24 ± 48039.43	-
	2 weeks	8.17 ± 0.93	<0.001	18.01 ± 1.55	<0.001	17.40 ± 1.07	0.04	164886.76 ± 34621.41	0.048
