# Supplementary material for: Skin Barrier-Enhancing Effects of Dermabiotics HDB with Regulation of Skin Microbiota
Source: J Microbiol Biotechnol. 2023 Oct 19;34(1):65–73. doi: 10.4014/jmb.2306.06042 (PMC10840481; doi:10.4014/jmb.2306.06042)
Supplement: Supplementary file 1 [file jmb-34-1-65-supple.pdf]

J. Microbiol. Biotechnol.
https://doi.org/10.4014/jmb.2306.06042

Supplementary table 1. Clinical data of skin parameters for each subjects

Sample*	Group	Moisture intensity (AU)		TEWL (g/m^2h)	Hot flush levels (pixels)
		Keratin layer surface	Keratin layer		
S01-L-0W	Test	4.74	7.79	19.27	505,710
S01-L-2W	Test	5.84	11.78	11.87	228,840
S01-R-0W	Control	4.61	6.62	20.20	480,090
S01-R-2W	Control	4.44	16.28	14.00	238,959
S02-L-0W	Test	6.19	15.30	22.20	415,700
S02-L-2W	Test	9.27	23.16	10.50	236,417
S02-R-0W	Control	2.92	6.44	20.93	983,301
S02-R-2W	Control	5.30	6.75	10.17	800,433
S03-L-0W	Control	5.00	12.49	25.17	243,446
S03-L-2W	Control	4.93	12.69	20.13	235,975
S03-R-0W	Test	4.38	7.42	24.97	482,252
S03-R-2W	Test	5.73	12.00	16.53	171,365
S04-L-0W	Control	3.73	8.81	26.37	15,253
S04-L-2W	Control	6.28	12.91	26.00	12,708
S04-R-0W	Test	3.24	7.96	29.00	7,414
S04-R-2W	Test	4.91	14.29	22.20	6,990
S05-L-0W	Control	5.49	22.38	20.23	560,176
S05-L-2W	Control	5.69	21.47	16.30	260,457
S05-R-0W	Test	2.65	4.84	22.07	689,035
S05-R-2W	Test	8.12	24.42	17.40	474,220
S06-L-0W	Control	6.92	7.58	37.33	257,336
S06-L-2W	Control	10.09	13.44	31.00	238,933
S06-R-0W	Test	5.18	11.27	37.00	159,306
S06-R-2W	Test	12.81	21.89	29.40	98,424
S07-L-0W	Test	4.99	8.41	18.30	9,222
S07-L-2W	Test	7.33	16.12	16.43	3,542
S07-R-0W	Control	4.22	7.48	16.30	100,702
S07-R-2W	Control	5.98	11.54	14.07	25,830
S08-L-0W	Control	4.02	20.04	30.70	8,308
S08-L-2W	Control	9.86	35.03	15.30	6,832
S08-R-0W	Test	5.51	15.87	23.43	6,117
S08-R-2W	Test	9.32	31.78	14.37	5,576
S09-L-0W	Control	3.16	12.37	25.80	474,308
S09-L-2W	Control	4.02	16.46	21.97	334,575
S09-R-0W	Test	3.59	11.01	22.87	746,636
S09-R-2W	Test	5.77	16.54	18.80	609,383
S10-L-0W	Control	3.71	6.37	24.67	257,694
S10-L-2W	Control	5.74	19.29	18.60	124,400
S10-R-0W	Test	3.01	4.39	18.67	419,033
S10-R-2W	Test	7.17	20.30	15.33	291,206
S11-L-0W	Test	3.93	11.15	32.67	154,632
S11-L-2W	Test	11.03	16.37	19.73	122,167
S11-R-0W	Control	3.56	8.91	16.87	24,845
S11-R-2W	Control	7.85	14.67	11.87	19,235
S12-L-0W	Control	2.46	14.89	28.57	124,038
S12-L-2W	Control	6.26	19.71	16.70	93,127

S12-R-0W	Test	3.19	18.34	21.70	439,908
S12-R-2W	Test	7.69	22.85	14.67	298,730
S13-L-0W	Test	2.47	6.41	27.73	185,258
S13-L-2W	Test	4.12	7.67	16.30	113,537
S13-R-0W	Control	2.64	7.44	24.23	211,394
S13-R-2W	Control	4.81	8.30	15.00	145,990
S14-L-0W	Control	9.30	23.50	23.03	31,706
S14-L-2W	Control	22.67	42.68	20.03	52,700
S14-R-0W	Test	8.57	14.37	25.60	26,382
S14-R-2W	Test	22.25	32.68	18.60	22,704
S15-L-0W	Test	4.96	9.26	23.93	225,678
S15-L-2W	Test	4.00	10.30	16.33	173,658
S15-R-0W	Control	3.50	5.70	16.37	139,399
S15-R-2W	Control	4.24	7.40	12.73	131,741
S16-L-0W	Control	2.67	4.21	16.27	5,933
S16-L-2W	Control	4.59	6.99	16.57	2,213
S16-R-0W	Test	2.41	2.91	20.33	62,478
S16-R-2W	Test	5.50	8.22	17.83	56,710
S17-L-0W	Test	4.26	22.00	21.63	76,706
S17-L-2W	Test	3.41	13.92	13.33	21,546
S17-R-0W	Control	3.77	11.95	20.63	283,088
S17-R-2W	Control	6.03	8.55	12.77	104,501
S18-L-0W	Test	2.45	4.78	11.80	304,235
S18-L-2W	Test	4.77	13.12	9.00	104,935
S18-R-0W	Control	2.75	4.04	13.17	634,121
S18-R-2W	Control	5.63	5.96	11.40	534,114
S19-L-0W	Control	2.42	6.83	31.30	304,186
S19-L-2W	Control	7.86	20.13	18.43	97,333
S19-R-0W	Test	2.41	4.03	30.07	330,717
S19-R-2W	Test	8.66	22.85	23.13	32,226
S20-L-0W	Test	5.90	14.17	28.83	311,556
S20-L-2W	Test	12.27	24.79	27.13	240,487
S20-R-0W	Control	5.54	10.78	26.07	486,473
S20-R-2W	Control	8.66	21.79	24.30	238,319
S21-L-0W	Test	2.55	6.01	23.53	371,537
S21-L-2W	Test	11.50	13.18	18.60	159,537
S21-R-0W	Control	2.76	5.20	24.23	690,873
S21-R-2W	Control	4.62	9.25	21.50	532,632
* 0W and 2W, before and after use of the cosmetic samples					

Supplementary table 2. Sequence quality statistics of each samples

Sample*	Group	Raw Reads	Total Bases (Mbp)	GC(%)	Q20(%)	Q30(%)	Processed Reads
S01-L-0W	Test	230,142	57.33	53.53	96.00	94.14	109,017
S01-L-2W	Test	219,420	54.63	54.12	96.05	94.16	101,036
S01-R-0W	Control	188,334	46.99	55.21	96.15	94.19	84,778
S01-R-2W	Control	206,890	51.61	55.80	95.97	93.97	91,172
S02-L-0W	Test	237,798	56.03	53.82	95.98	94.16	105,418
S02-L-2W	Test	244,870	54.81	50.68	92.17	89.07	90,168
S02-R-0W	Control	197,910	47.12	53.80	95.29	93.19	88,729
S02-R-2W	Control	235,638	55.68	53.80	95.78	93.94	104,007
S03-L-0W	Control	207,656	51.11	54.59	97.10	95.70	98,290
S03-L-2W	Control	187,778	46.54	56.33	97.43	96.14	89,697
S03-R-0W	Test	224,434	55.53	54.41	96.74	95.20	106,362
S03-R-2W	Test	238,136	58.52	55.57	96.24	94.52	111,096
S04-L-0W	Control	210,376	50.10	53.51	96.09	94.24	94,302
S04-L-2W	Control	189,974	46.05	55.01	96.36	94.62	86,884
S04-R-0W	Test	215,144	51.55	53.38	95.36	93.21	98,905
S04-R-2W	Test	217,828	52.76	54.86	96.89	95.33	101,148
S05-L-0W	Control	224,548	52.99	54.18	95.57	93.57	95,786
S05-L-2W	Control	174,504	42.44	55.99	95.58	93.50	78,257
S05-R-0W	Test	208,546	50.59	55.78	95.90	93.93	93,122
S05-R-2W	Test	193,908	47.03	55.84	96.65	94.94	87,812
S06-L-0W	Control	209,230	51.70	55.73	97.10	95.64	99,501
S06-L-2W	Control	231,618	57.47	55.85	97.21	95.78	109,764
S06-R-0W	Test	244,456	60.19	55.77	97.05	95.57	116,782
S06-R-2W	Test	206,822	50.85	55.08	97.15	95.63	97,351
S07-L-0W	Test	236,628	57.31	55.40	96.35	94.63	108,781
S07-L-2W	Test	194,086	46.80	55.19	96.40	94.72	89,890
S07-R-0W	Control	236,800	57.21	55.12	96.54	94.97	108,934
S07-R-2W	Control	207,972	50.17	55.07	96.33	94.67	93,890
S08-L-0W	Control	234,336	58.29	53.32	97.32	96.08	113,808
S08-L-2W	Control	235,246	56.34	53.88	97.04	95.64	111,616
S08-R-0W	Test	249,620	61.36	56.09	97.12	95.72	119,633
S08-R-2W	Test	234,130	57.15	55.06	97.06	95.61	110,217
S09-L-0W	Control	225,548	53.62	54.51	96.49	94.85	104,006
S09-L-2W	Control	219,530	50.78	53.01	94.95	92.86	92,649
S09-R-0W	Test	240,590	57.02	54.41	95.99	94.20	108,943
S09-R-2W	Test	230,058	54.72	54.66	96.43	94.80	103,739
S10-L-0W	Control	203,748	50.21	56.33	96.85	95.31	97,476
S10-L-2W	Control	240,862	59.80	56.67	97.32	95.96	117,012
S10-R-0W	Test	213,724	52.27	55.77	96.93	95.42	100,322
S10-R-2W	Test	224,044	54.28	55.45	97.24	95.87	105,029
S11-L-0W	Test	226,778	55.91	53.13	96.40	94.65	107,436
S11-L-2W	Test	250,866	62.05	53.42	96.89	95.37	119,635
S11-R-0W	Control	241,802	58.61	51.82	96.02	94.15	110,864
S11-R-2W	Control	223,522	55.20	53.53	96.60	94.95	106,050
S12-L-0W	Control	221,082	53.72	52.82	96.24	94.58	99,901
S12-L-2W	Control	225,286	55.94	54.16	96.87	95.40	108,682
S12-R-0W	Test	195,280	47.55	53.44	95.58	93.66	88,919
S12-R-2W	Test	215,984	53.14	54.21	97.20	95.86	103,302
S13-L-0W	Test	215,518	51.79	54.11	95.91	94.08	95,328
S13-L-2W	Test	249,426	61.96	55.71	97.60	96.39	120,351

S13-R-0W	Control	250,134	59.79	53.58	96.25	94.56	111,012
S13-R-2W	Control	206,194	51.61	56.60	96.99	95.52	100,437
S14-L-0W	Control	270,120	66.11	55.44	96.85	95.26	128,760
S14-L-2W	Control	227,262	54.94	55.34	96.35	94.62	104,945
S14-R-0W	Test	231,100	55.93	54.62	96.58	94.86	110,007
S14-R-2W	Test	242,252	57.80	54.55	96.53	94.87	113,334
S15-L-0W	Test	230,582	54.82	54.51	96.19	94.35	104,395
S15-L-2W	Test	188,388	44.86	54.73	96.13	94.26	86,775
S15-R-0W	Control	215,998	52.56	55.96	96.38	94.57	98,908
S15-R-2W	Control	218,452	51.76	54.60	95.82	93.78	97,825
S16-L-0W	Control	242,792	59.22	55.25	96.83	95.28	115,122
S16-L-2W	Control	275,420	64.99	53.82	95.91	94.09	124,913
S16-R-0W	Test	238,022	57.81	54.44	96.14	94.31	108,761
S16-R-2W	Test	249,248	60.64	54.97	96.59	94.93	116,355
S17-L-0W	Test	232,110	56.47	55.67	95.86	93.89	107,007
S17-L-2W	Test	215,594	52.40	56.04	96.65	94.98	97,830
S17-R-0W	Control	206,882	50.29	55.81	96.22	94.36	94,373
S17-R-2W	Control	202,136	49.72	56.80	96.09	94.12	90,788
S18-L-0W	Test	227,340	54.01	54.10	95.93	94.08	105,925
S18-L-2W	Test	246,150	60.31	55.47	97.03	95.55	116,194
S18-R-0W	Control	207,884	50.12	54.17	95.45	93.37	94,583
S18-R-2W	Control	228,146	55.08	55.27	96.13	94.33	104,750
S19-L-0W	Control	229,310	55.61	54.35	95.89	93.90	104,051
S19-L-2W	Control	218,342	53.10	55.19	96.35	94.57	101,215
S19-R-0W	Test	216,942	51.99	54.95	94.70	92.32	94,026
S19-R-2W	Test	271,194	65.47	55.38	96.38	94.64	122,379
S20-L-0W	Test	194,414	47.58	54.10	95.38	93.33	89,419
S20-L-2W	Test	228,610	55.94	54.57	96.17	94.34	108,538
S20-R-0W	Control	211,304	52.23	54.56	96.71	95.14	97,696
S20-R-2W	Control	230,712	55.80	53.90	96.12	94.36	105,287
S21-L-0W	Test	211,538	51.08	54.63	95.56	93.53	95,725
S21-L-2W	Test	217,784	52.71	54.92	96.68	95.04	101,210
S21-R-0W	Control	236,780	56.97	55.00	96.45	94.75	109,046
S21-R-2W	Control	198,564	48.13	53.17	94.82	92.30	90,134
* 0W and 2W, before and after use of the cosmetic samples							

Supplmementary figure 1. β-diversity with Principal coordinates analysis (PCoA) plot of unweighted UniFrac distances and canonical correspondence analysis (CCA).

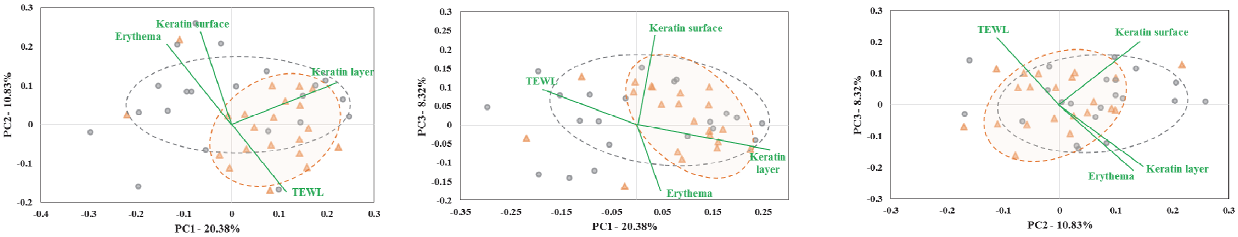

* Grey circles and orange triangles represent control and HDB before use each.
